# Combination of Tumor Mutational Burden and Specific Gene Mutations Stratifies Outcome to Immunotherapy Across Recurrent and Metastatic Head and Neck Squamous Cell Carcinoma

**DOI:** 10.3389/fgene.2021.756506

**Published:** 2021-11-16

**Authors:** Ying-Peng Peng, Rong Wang, Qiao-Dan Liu, Xi-Wei Xu, Wei Wei, Xiao-Tao Huang, Xiao-Mou Peng, Zhi-Gang Liu

**Affiliations:** ^1^ The Cancer Center of the Fifth Affiliated Hospital of Sun Yat-sen University, Zhuhai, China; ^2^ Guangdong Provincial Key Laboratory of Biomedical Imaging, The Fifth Affiliated Hospital, Sun Yat-sen University, Zhuhai, China; ^3^ Center of Infectious Diseases, The Fifth Affiliated Hospital, Sun Yat-Sen University, Zhuhai, China

**Keywords:** tumor mutational burden, gene mutation, immunotherapy, survival prognosis, recurrent and metastatic head and neck squamous cell carcinoma

## Abstract

**Purpose:** To investigate the prognostic significance of tumor mutational burden (TMB) combined with specific prognosis-related gene mutations in immunotherapy for recurrent and metastatic head and neck squamous cell carcinoma (r/m HNSCC).

**Methods:** One hundred thirty-two r/m HNSCC patients from the Morris and Allen cohorts had undergone immunotherapy. We constructed the immunotherapy-related gene prognostic index TP-PR combining TMB and *PIK3CA*, *TP53*, or *ROS1* mutation. And we analyzed the differences in overall survival (OS) and immune cell infiltration between samples in different groups. The association of each signature’s single-sample gene set enrichment analysis scores with TP-PR was tested using Spearman’s correlation test.

**Results:** The median OS of the patients with high TMB (TMB ≥10 mut/Mb) who received immunotherapy for r/m HNSCC was 2.5 times as long as that of the patients with low TMB (25 vs. 10 months). More importantly, the high TP-PR (TP-PR >0) group had better median OS (25 vs. 8 months) than the low TP-PR (TP-PR ≤0) group. CD8^+^ T cells and activated memory CD4^+^ T cells in the tissues of the patients with high TP-PR were higher than those in the patients with low TP-PR. Results showed that TP-PR stratification had a higher area under the curve (AUC) value (0.77, 95% CI 0.86–0.68) compared with TMB stratification (0.56, 95% CI 0.68–0.44). The differential gene expression in the high and low TP-PR groups mainly influenced metabolism-related signaling pathways.

**Conclusion:** TP-PR was an effective predictor of immunotherapy outcome for r/m HNSCC, which might be better than TMB alone. Patients with high TP-PR had a better survival benefit than had the patients with low TP-PR.

## Introduction

Head and neck squamous cell carcinoma (HNSCC) is a general term for squamous cell carcinoma originating in the head and neck, including the oral cavity, nasopharynx, oropharynx, hypopharynx, and larynx (Johnson et al., 2020). The incidence of HNSCC is the sixth highest in the world, with more than 830,000 people diagnosed each year and a fatality rate of approximately 50% (Bray et al., 2018). At present, the first-line treatment for HNSCC is mainly surgery, radiotherapy, and chemotherapy. Recurrent and metastatic (r/m) HNSCC has been treated with platinum-based chemotherapy in combination with an anti-epidermal growth factor receptor targeted drug cetuximab (Leemans et al., 2018). However, no matter what chemotherapy regimen was used in combination with cetuximab, the median overall survival (OS) of r/m HNSCC patients was approximately 10 months (Zhu et al., 2021). KEYNOTE-048 was a landmark clinical study performed for r/m HNSCC treatment, which demonstrated that the first-line treatment of r/m HNSCC population with positive programmed cell death ligand 1 (PD-L1) expression with pembrolizumab alone or combined with platinum-containing chemotherapy presented a greater survival benefit than traditional targeted first-line chemotherapy drugs (Burtness et al., 2019). The approval of pembrolizumab as a first-line therapy for r/m HNSCC marks the beginning of the era of immunotherapy for head and neck tumors.

However, one of the major limitations of immune checkpoint inhibitor (ICI) treatment is its low response rate. Therefore, we need to find more valuable biomarkers to predict the effect of immunotherapy on the prognosis of patients with r/m HNSCC. KEYNOTE-158 clinical trial was performed for advanced solid tumors including anal, biliary, and 10 other cancers (HNSCC was not included). It demonstrated significant improvement in objective response rate and progression-free survival after immunotherapy in patients with tumor mutational burden (TMB) ≥10 mut/Mb (Marabelle et al., 2020). As a result of this study, TMB became the second approved “pan-cancer” biomarker following microsatellite instability (Goodman et al., 2017; Marabelle et al., 2020; Valero, 2021).

The role of TMB in predicting the outcome of immunotherapy for advanced HNSCC remains unclear because of absence of definitive data. A previous study showed that TMB is a predictor of prognosis in patients with HNSCC, and high TMB (TMB-H) (top 25%) has a greater survival benefit than low TMB (TMB-L) (bottom 25%), whereas TMB ≥10 mut/Mb was not prognostic for them (Zhang et al., 2020a). But they did not investigate the prognosis of immunotherapy efficacy. Another study showed that microsatellite-stable solid tumors with TMB-H (TMB ≥10 mut/Mb) had higher response rates than those with TMB-L after immunotherapy (Valero, 2021). However, there were no data on survival prognosis.

To determine the significance of TMB in predicting immunotherapy outcome in patients with r/m HNSCC, and to find a valuable prognostic index, we retrospectively analyzed survival data, TMB expression, and gene mutations in patients with r/m HNSCC using The Cancer Genome Atlas (TCGA) and the cBioPortal databases. We further constructed the immunotherapy-related gene prognostic index TP-PR for r/m HNSCC to clarify the benefits of ICI therapy for some TP-PR-defined group.

## Methods

### Patient Selection

**FIGURE 1:**
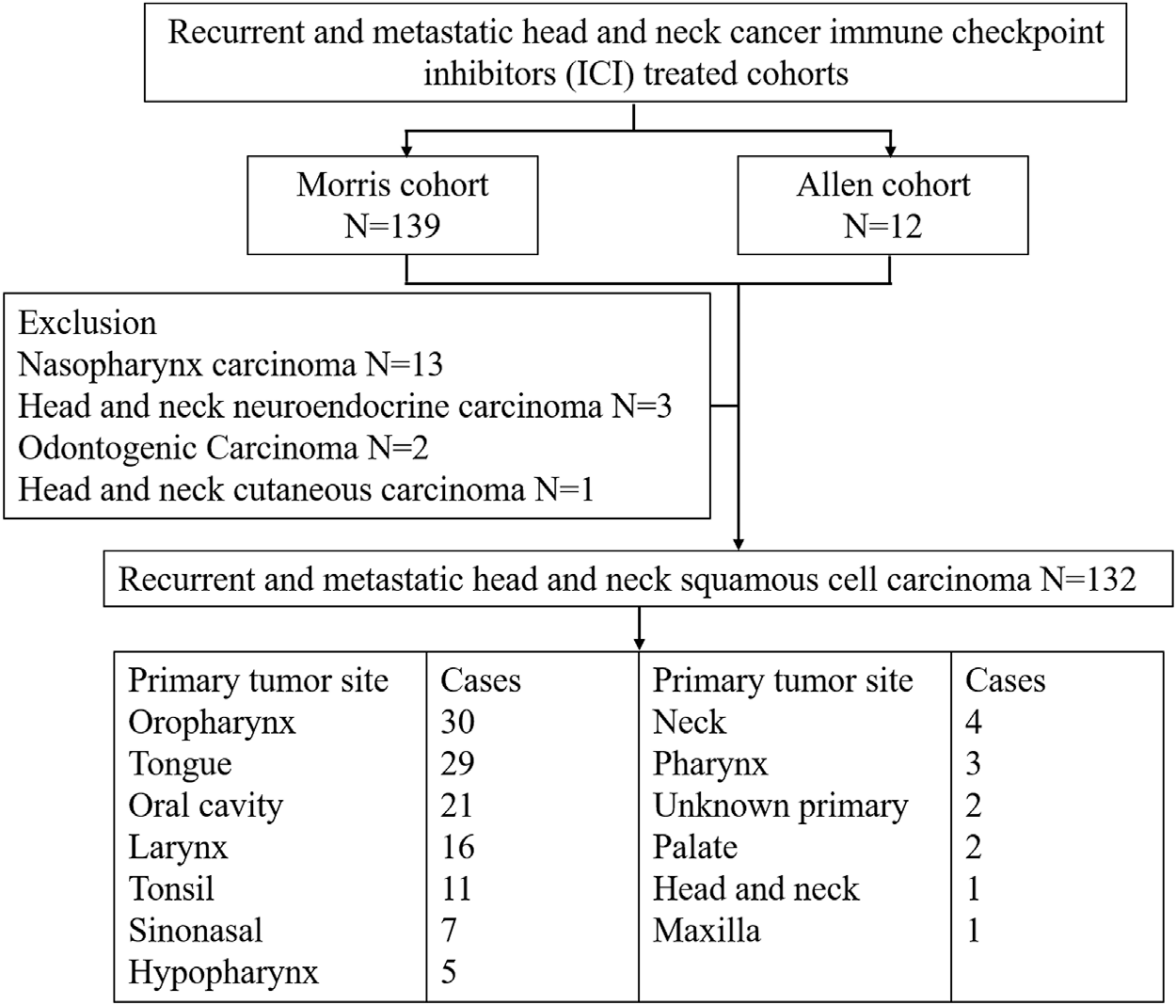
Patient selection of recurrent and metastatic head and neck cancer immune checkpoint inhibitor (ICI)-treated cohorts.

We used five cohorts, namely, the Morris cohort (Samstein et al., 2019), the Allen cohort (Miao et al., 2018), the Ho cohort (Morris et al., 2017), the Berger cohort (Zehir et al., 2017), and TCGA cohort. The inclusion criteria for r/m HNSCC is shown in Figure 1. The Morris cohort finally included 120 patients, and the Allen cohort included 12 patients (Supplemental Table S1). All patients were diagnosed with r/m HNSCC, not including nasopharyngeal carcinoma. Those 132 patients had undergone immunotherapy with anti-programmed cell death protein 1 (PD1)/PD-L1 agents alone or combined with CTLA-4 inhibitors. Only patients for whom whole-exome sequencing (WES) and survival information were available were analyzed for this study. The Ho cohort included 115 r/m HNSCC patients, and the Berger cohort included 49 r/m HNSCC patients who did not receive immunotherapy (Supplemental Table S2, S3). TCGA cohort included 488 patients with HNSCC with few r/m HNSCC patients included (Supplemental Table S4). No patient from TCGA cohort had received immunotherapy. All relevant information from the five cohorts was downloaded from cbioportal.org, including mutation landscape data, WES, and RNA sequencing (RNA-seq) data, and survival information.

### Tumor Mutational Burden Score

TMB was calculated as total variants divided by the total callable bases covered by the MSK-IMPACT or WES panel in megabases (Mb) for both the intronic and exonic sequences (total variants/Mb) or for exonic and splice site sequence (nonsynonymous mutations/Mb). TMB score of the Morris cohort, the Allen cohort, the Ho cohort, and the Berger cohort was downloaded from cbioportal.org. TMB score of TCGA cohort was downloaded from the literature (Thorsson et al., 2018). The mean coverage of the WES was 38 Mb. Variant allele frequencies cutoffs can vary from 0.5 to 10%, with lower thresholds increasing the risk of including false positives arising from sequencing artifacts such as C-to-T transitions introduced by formalin fixation (Chan et al., 2019). Thus, we selected variants with an allelic fraction of at least 10%.

### Correlation Analysis of Tumor Immunogenicity and Immune Characteristics

We used the CIBERSORT web portal (https://cibersort.stanford.edu/) to evaluate the infiltration status of 22 immune cell types for TCGA cohort. Then, we analyzed the differences in immune cell infiltration between the samples in different groups.

### RNA Sequencing Data Processing and Analysis

RNA-seq data were downloaded from UCSC Xena (https://xenabrowser.net/datapages). RNA-seq count data normalized to FPKM (fragments per kilobase per million) values were used as input for single-sample gene set enrichment analysis (ssGSEA). ssGSEA was performed for select signatures using the gene set variation analysis package with default settings. The association of each signature’s ssGSEA scores with TP-PR was tested using Spearman’s correlation test.

### Construction of a Prognostic Model (TP-PR Score)

Univariate and multivariate Cox regression analyses were performed to analyze the gene mutations significantly associated with OS. TMB and the gene mutations significantly affecting OS were used to construct TP-PR score. The TP-PR score of each sample was calculated by multiplying the values of certain indexes by their weight in the Cox model and then adding them together. The prognostic index TP-PR (formula: TP-PR = TMB * 1.0803 + *PIK3CA* * 0.654 − *TP53* * 0.5581 − *ROS1* * 1.2342) was then constructed according to the expression of those three genes. It was scored 1 if TMB ≥10 mut/Mb or if the gene was mutated and was scored 0 if TMB <10 mut/Mb or if the gene was wide-type. The final score was calculated as the TP-PR score. Regression coefficients were generated in the process of multifactor Cox regression analysis with R software (version 4.1.1). The prognostic power of the TP-PR score was evaluated by Kaplan–Meier (K-M) survival curves with log-rank tests with the ICI cohort.

### Statistics

The K-M estimator was used to describe the distribution of survival time. The log-rank Mantel–Cox test was used to compare survival between different strata. Statistical analyses were performed using GraphPad software, version 8.0 (GraphPad Software Inc., La Jolla, CA, USA). All data points and statistical analyses represent individual patients.

## Results

### Tumor Mutational Burden is a Prognostic Factor for the Overall Survival of Recurrent and Metastatic Head and Neck Squamous Cell Carcinoma Patients Undergoing Immunotherapy

**FIGURE 2:**
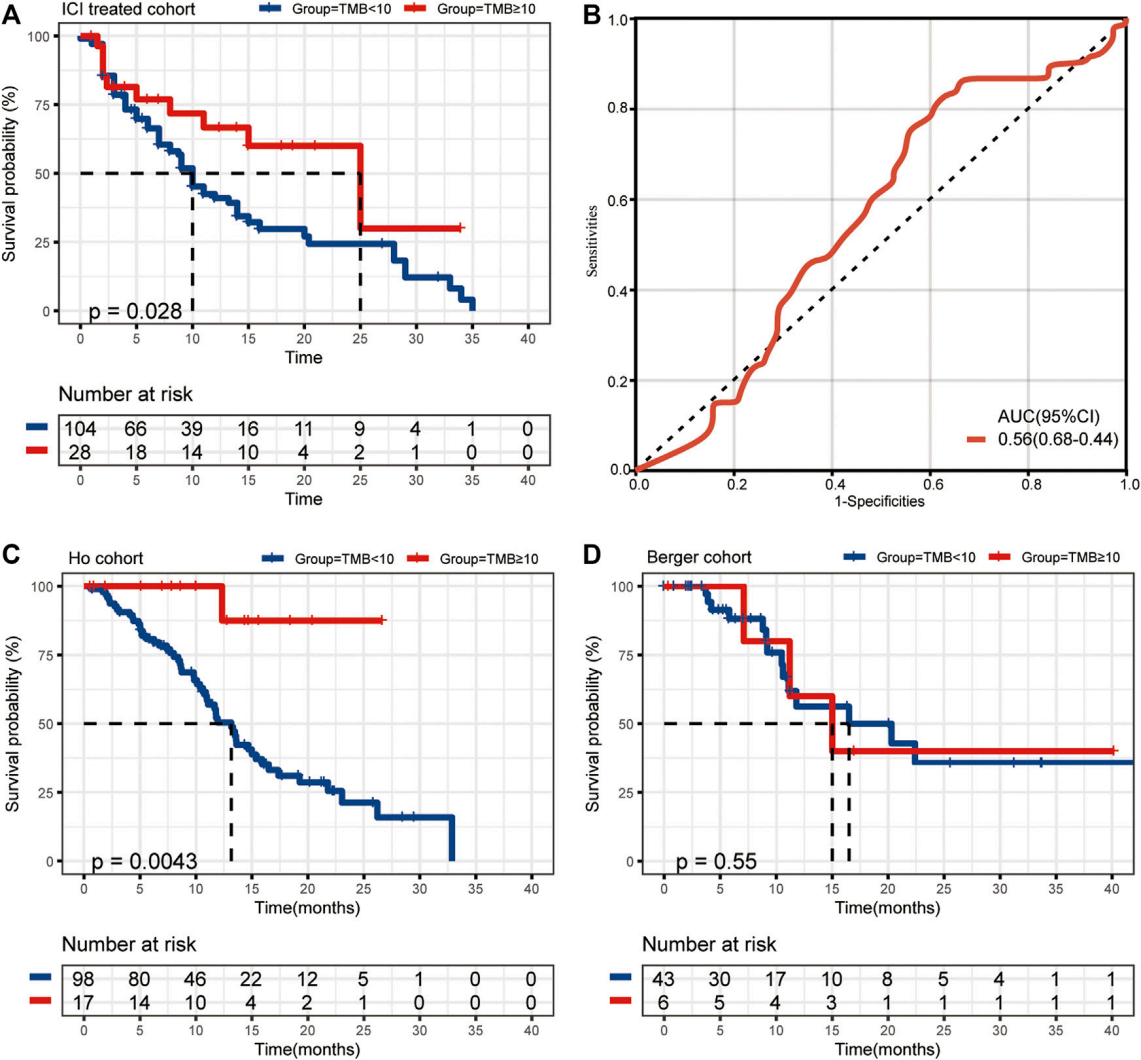
**(A)** The median OS of ICI-treated r/m HNSCC patients with high and low TMB (25 vs*.* 10 months, *p* = 0.028). **(B)** The ROC curve of TMB stratification. **(C)** The difference in median OS for non-ICI-treated HNSCC patients with high and low TMB (the Ho cohort, *p* = 0.0043). **(D)** The difference in median OS for non-ICI-treated HNSCC patients with high and low TMB (the Berger cohort, *p* = 0.55). OS, overall survival; TMB, tumor mutational burden; ICI, immune checkpoint inhibitor; r/m HNSCC, recurrent and metastatic head and neck squamous cell carcinoma; ROC, receiver operating characteristic.

We collected the data of 132 r/m HNSCC patients receiving immunotherapy from cBioPortal (https://www.cbioportal.org/study/summary?id=tmb_mskcc_2018; https://www.cbioportal.org/study/summary?id=mixed_allen_2018). The inclusion criteria for r/m HNSCC are shown in Figure 1. Detailed characteristics including age, gender, sample type, and administered immunotherapy drugs are shown in Table 1. We characterized patients as having TMB-H or TMB-L on the basis of the threshold of TMB ≥10 mut/Mb. Our analysis showed that the median OS of patients with TMB-H was 2.5 times as long as that of the patients with TMB-L (25 vs. 10 months), with an area under the curve (AUC) value of 0.56 (95% CI 0.68–0.44) (Figures 2A,B). In other words, immunotherapy was more effective in r/m HNSCC patients with TMB-H (TMB ≥10 mut/Mb). In addition, we collected data from two other cohorts that included r/m HNSCC patients receiving no immunotherapy (Figures 2C,D). Results showed that the difference in median OS between the patients receiving no immunotherapy with TMB-H and those with TMB-L was significant in the Ho cohort (Figure 2C, *p* = 0.0043) and not significant in the Berger cohort (Figure 2D, *p* = 0.55). It reminded us that the significance of TMB stratification in non-ICI cohorts of HNSCC was still uncertain. Thus, we need to look for a comprehensive prognostic index other than TMB alone.

**TABLE 1:** Overview of ICI-treated cohort used in this study.

	ICI-treated cohorts	Uni Cox	Multi Cox
**TMB**			
<10	104	0.028	0.042
≥10	28		
**Age**			
≤65	70	0.045	0.104
>65	50		
NA	12		
**Sex**			
Female	25	0.312	**\**
Male	107		
**Drug type**			
Combo	11	0.343	**\**
PD-1/PD-L1	121		
***TP53***			
Mutation	59	0.072	0.012
Wild type	73		
***PIK3CA***			
Mutation	28	0.008	0.019
Wild type	104		
***ROS1***			
Mutation	6	0.038	0.018
Wild type	126		
***ARID1A***			
Mutation	9	0.092	0.114
Wild type	123		
***ATR***			
Mutation	7	0.074	0.097
Wild type	125		
***ASXL1***			
Mutation	7	0.094	0.174
Wild type	125		

Note. ICI, immune checkpoint inhibitor; TMB, tumor mutational burden.

### Construction of TP-PR Model

**FIGURE 3:**
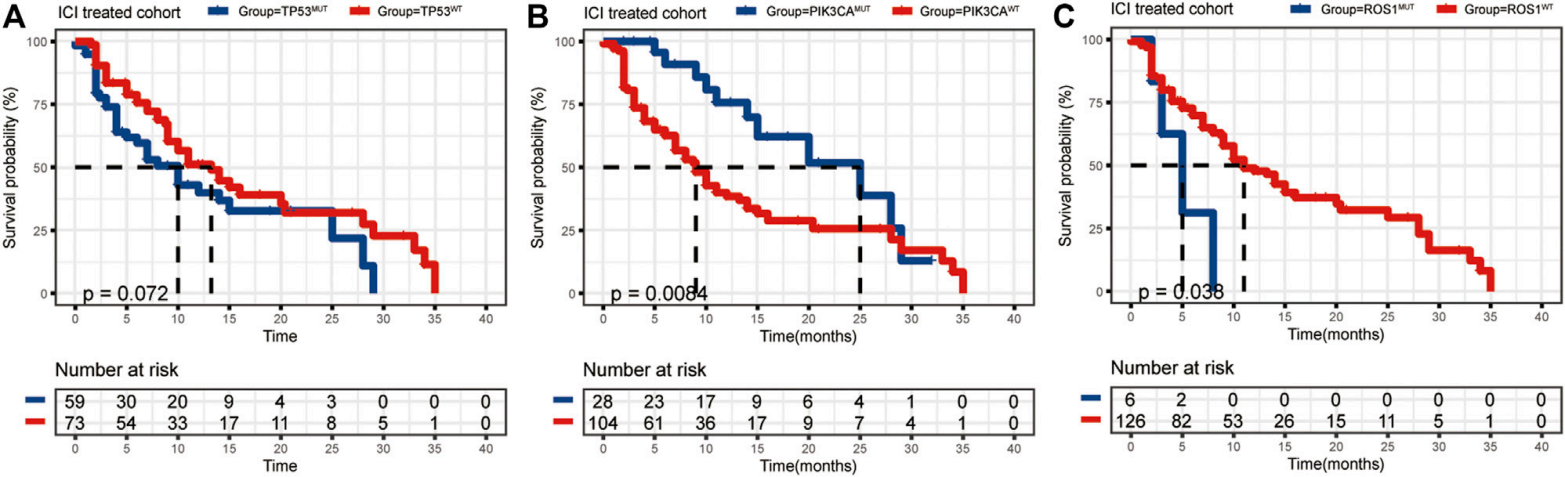
**(A–C)** K-M analysis of ICI-treated r/m HNSCC patients with *TP53*, *PIK3CA*, and *ROS1* mutations. K-M, Kaplan–Meier; ICI, immune checkpoint inhibitor; r/m HNSCC, recurrent and metastatic head and neck squamous cell carcinoma.

**FIGURE 4:**
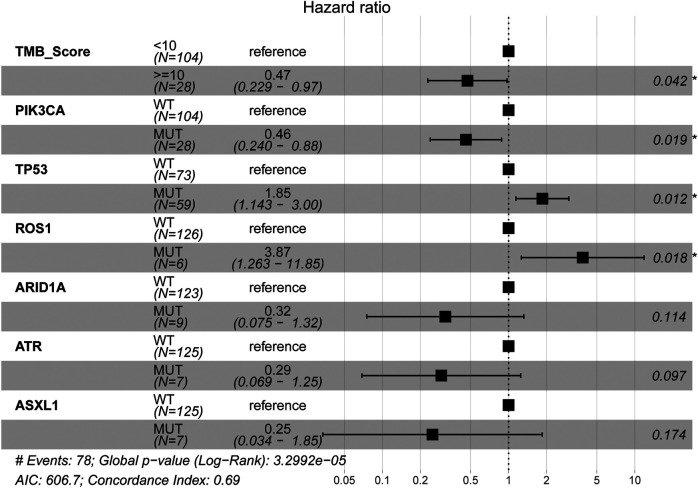
Multivariate Cox regression analysis of TMB and six genes, *PIK3CA*, *TP53*, *ARID1A*, *ROS1*, *ATR*, and *ASXL1*. TMB, tumor mutational burden.

The mutational landscape showed that the commonly mutated genes in r/m HNSCC patients from ICI-treated cohorts were *TP53*, *TERT*, *PIK3CA*, *NOTCH1*, *FAT1*, *KMT2D*, and *CDKN2A* (Supplementary Figure S1A). The flowchart of gene selection is shown in Supplementary Figure S1B. Twenty-seven genes with mutation frequency of no less than 5% were obtained from the results of mutational landscape. The correlation between those gene mutations and the OS was analyzed by K-M univariate analysis (Figures 3A–C; Supplementary Figure S2A–X). K-M univariate analysis showed that *TP53*, *PIK3CA*, *ARID1A*, *ROS1*, *ATR*, and *ASXL1* might be related to the OS (*p* < 0.1) for r/m HNSCC patients who received immunotherapy. Then the six mutation genes and TMB were included for multivariate Cox regression analysis. Finally, the three genes, *PIK3CA*, *TP53*, and *ROS1*, were found to be associated with the OS (*p* < 0.05) (Figure 4). Among them, TMB-H or *PIK3CA* mutation was positively correlated with OS, while *TP53* or *ROS1* mutation was negatively correlated with OS. The prognostic index TP-PR (formula: TP-PR = TMB * 1.0803 + *PIK3CA* * 0.654 − *TP53* * 0.5581 − *ROS1* * 1.2342) was then constructed according to the expression of those three genes. It was scored 1 if TMB ≥10 mut/Mb or if the gene was mutated and was scored 0 if TMB <10 mut/Mb or if the gene was wide-type. The final score was calculated as the TP-PR score.

### TP-PR is Better Than Tumor Mutational Burden Alone to Screen Out More Recurrent and Metastatic Head and Neck Squamous Cell Carcinoma Patients Who Might Benefit From Immunotherapy

**FIGURE 5:**
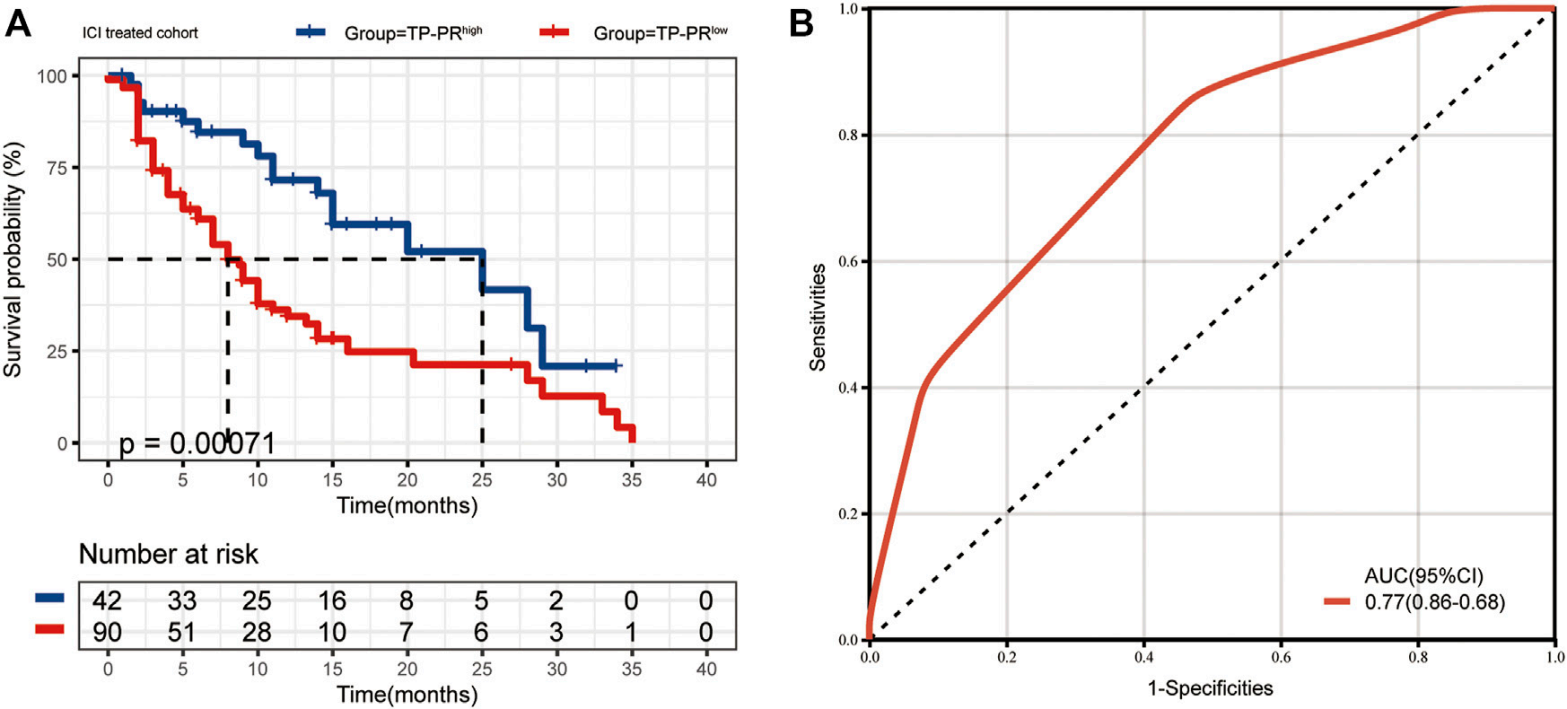
**(A)** The median OS of ICI-treated r/m HNSCC patients with high TP-PR and low TP-PR (25 vs. 8 months, *p* = 0.00071). **(B)** The ROC curve of TP-PR stratification. OS, overall survival; ICI, immune checkpoint inhibitor; r/m HNSCC, recurrent and metastatic head and neck squamous cell carcinoma; ROC, receiver operating characteristic.

**FIGURE 6:**
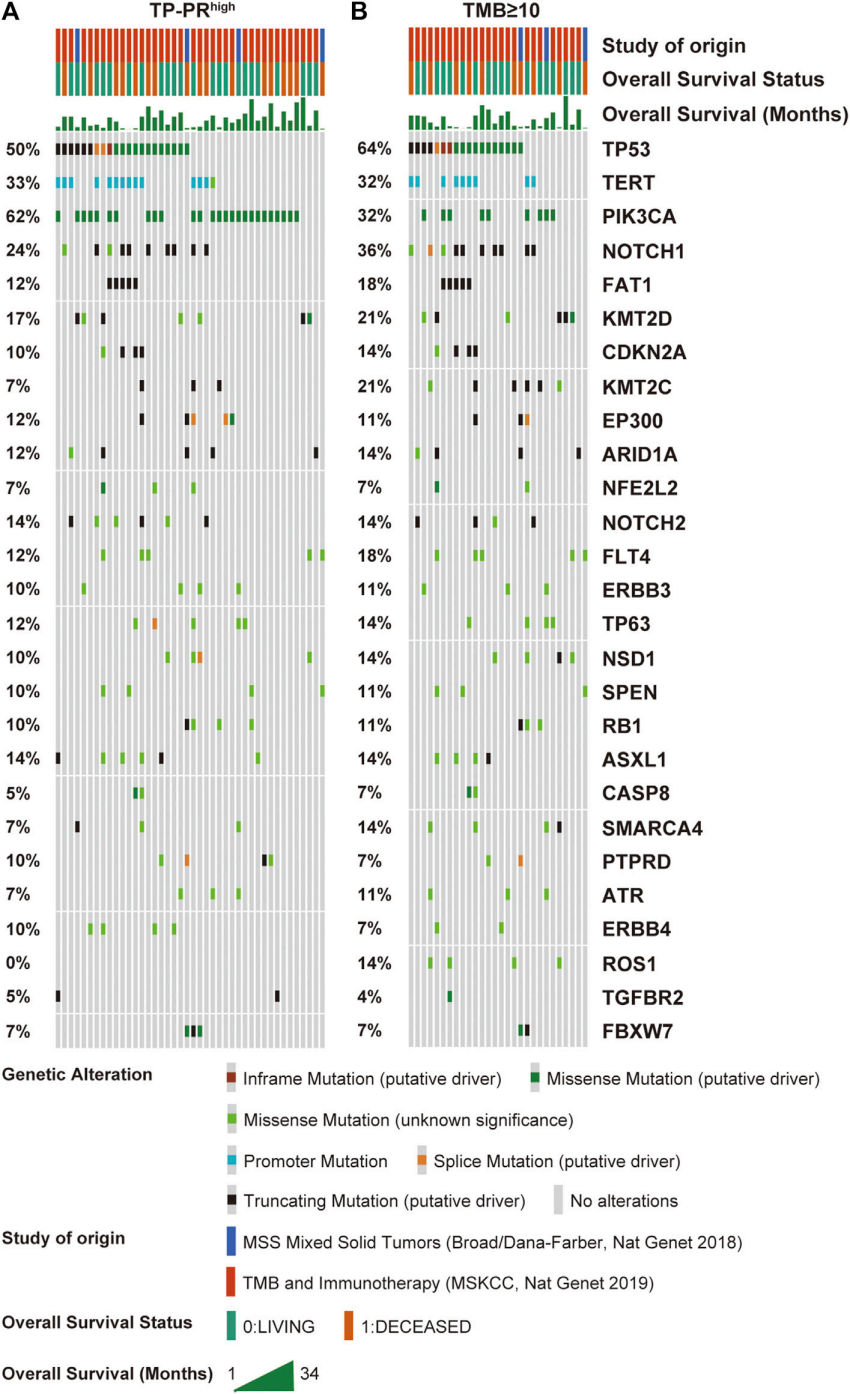
Mutational landscape of high TP-PR vs. high TMB in ICI-treated r/m HNSCC patients. Gene mutations **(A)** and their proportions **(B)** are shown. Specific mutation types are displayed in different colors and shown in the bottom panel. TMB, tumor mutational burden; ICI, immune checkpoint inhibitor; r/m HNSCC, recurrent and metastatic head and neck squamous cell carcinoma.

The data sets of r/m HNSCC patients who had received immunotherapy were divided into two groups: high TP-PR (TP-PR >0) and low TP-PR (TP-PR ≤0). We found that the high TP-PR group had better median OS (25 vs. 8 months) than the low TP-PR group, with a higher AUC value (0.77, 95% CI 0.86–0.68) than TMB stratification (Figures 5A,B). Moreover, the high TP-PR group screened out more people than the TMB-H group. In other words, TP-PR screened out more people who might benefit from immunotherapy than TMB. We further contrasted the mutational landscape of TP-PR high population and TMB-H population (Figure 6). We found that *PIK3CA* mutation rate was higher in the TP-PR high population, whereas *TP53* and especially *ROS1* (0 vs. 14%) mutation rates were higher in the TMB-H population. That was in accordance with our conclusion that *PIK3CA* mutation was positively correlated with OS, while *TP53* or *ROS1* mutation was negatively correlated with OS. And it might be the answer why TP-PR screened out more people who might benefit from immunotherapy than TMB alone. Gene mutation analysis was also performed between the high and low TP-PR groups. It was confirmed that the expressions of *PIK3CA*, *TP53*, and *ROS1* were different in the high and low TP-PR groups; especially, those of *PIK3CA* and *ROS1* were significantly different (data not shown). It supported that *PIK3CA*, *TP53*, and *ROS1* were related to the prognosis of r/m HNSCC patients.

### Analysis of Immune Infiltration and Pathway Enrichment in the Cancer Genome Atlas Cohort

**FIGURE 7:**
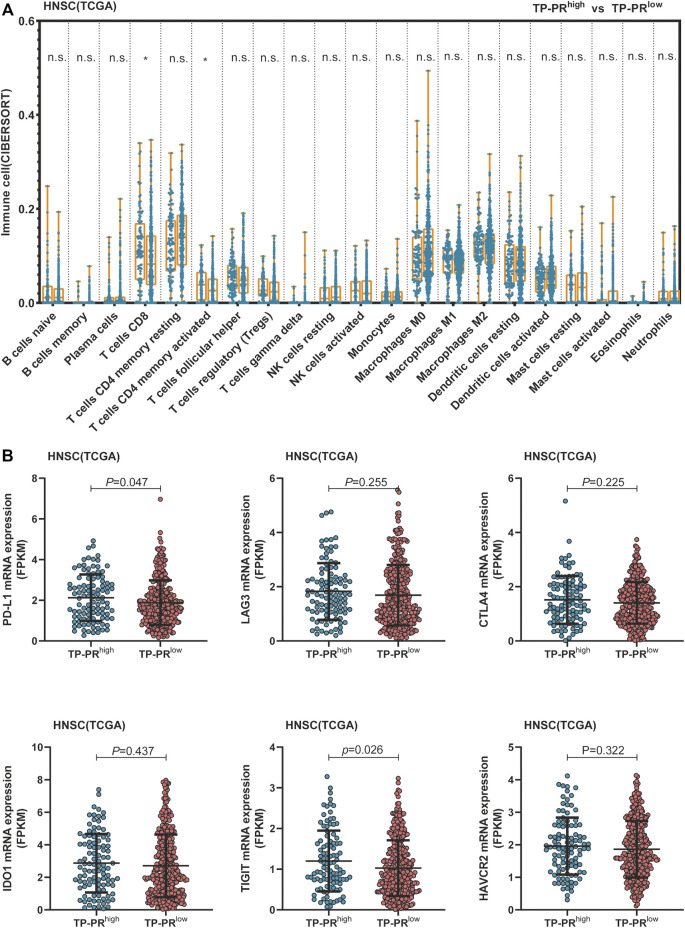
**(A)** Comparison of immune cell infiltration between the high and low TP-PR HNSCC patients in TCGA cohort. **(B)** mRNA expression of some immunotherapy-related targets in high TP-PR vs. low TP-PR HNSCC patients. HNSCC, head and neck squamous cell carcinoma; TCGA, The Cancer Genome Atlas.

TCGA cohort included 488 patients with HNSCC who had not received immunotherapy. RNA-seq analysis of TCGA cohort showed that CD8^+^ T cells and activated memory CD4^+^ T-cell infiltration in the tissues of the patients with high TP-PR were higher than those in the patients with low TP-PR, and the differences were statistically significant (Figure 7A). Infiltration of other immune cells was not significantly different between the two groups. It was suggested that high TP-PR might enhance CD8^+^ T cells and activated memory CD4^+^ T-cell infiltration, without affecting the mRNA expression of immunotherapy-related targets like LAG3, CTLA4, IDO1, and HAVCR2 (Figure 7B). But the PD-L1 (*p* = 0.047) and TIGIT (*p* = 0.026) mRNA expressions were higher in the high TP-PR group than in the low TP-PR group, indicating that high TP-PR might promote the mRNA expression of PD-L1 and TIGIT.

**FIGURE 8:**
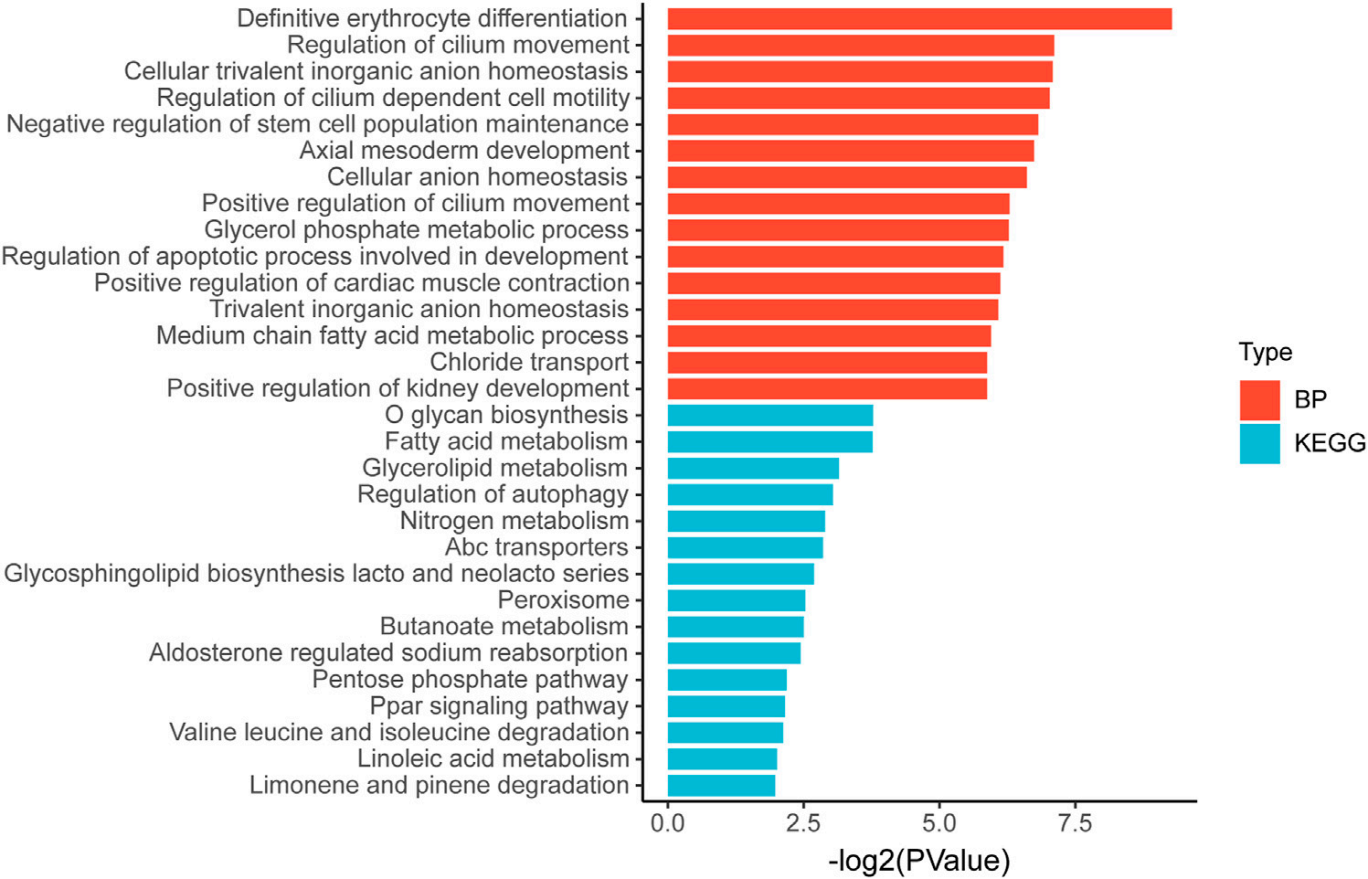
Gene ontology and Kyoto Encyclopedia of Genes and Genomes pathway enrichment analysis of differential gene expression in high and low TP-PR groups.

Then we tried to find biological processes (BPs) and signaling pathways that might be relevant by gene ontology and Kyoto Encyclopedia of Genes and Genomes analyses. Figure 8 shows the 15 most relevant BPs or signaling pathways. It is suggested that the differential gene expression in the high and low TP-PR groups in TCGA cohort mainly influenced BP related to development and metabolism, and metabolism-related signaling pathways, such as the pathways for fatty acid metabolism, glycerolipid metabolism, and nitrogen metabolism.

RNA-seq analysis also showed that CD8^+^ T cells and resting memory CD4^+^ T-cell infiltration in patients with TMB-H were higher than those in patients with TMB-L, with a statistically significant difference (Supplementary Figure S3A). But there was no difference in the mRNA expression of PD-L1, TIGIT, LAG3, CTLA4, IDO1, and HAVCR2 between the TMB-H and TMB-L groups (Supplementary Figure S3B). Compared with analysis stratified by TMB alone, patients with TP-PR stratification showed a difference in the mRNA expression of PD-L1 and TIGIT, which might influence the immune microenvironment. The differential gene expression in TMB-H and TMB-L groups in TCGA cohort also mainly influenced metabolism-related signaling pathways (Supplementary Figure S4).

## Discussion

With immunotherapy now being included in the first-line treatment regimen for r/m HNSCC, finding appropriate biomarkers for predicting the efficacy of immunotherapy has become an urgent problem that needs to be solved. Positive expression of PD-L1 [combined positive score (CPS) ≥1] has become a concomitant indication for the use of pembrolizumab in HNSCC. The role of TMB in predicting the immunotherapy efficacy for r/m HNSCC has also not been well determined. Our analysis showed that immunotherapy was more effective in r/m HNSCC patients with TMB-H (TMB ≥ 10 mut/Mb), and the median OS of these patients was 2.5 times as long as that of the patients with TMB-L (25 vs. 10 months). However, the significance of TMB stratification in non-ICI cohorts of HNSCC was still uncertain. Although current evidence suggests that TMB is associated with increased efficacy of ICIs (Chen et al., 2020; Sha et al., 2020), the underlying mechanism linking TMB to immunotherapy benefits is not fully understood (Dos Santos et al., 2021). In addition, some studies have suggested that TMB is only useful for predicting the clinical response of some cancer patients (those with endometrial cancer, colorectal cancer, melanoma, lung adenocarcinoma, etc.) to ICIs, and some solid tumors with TMB-H failed to achieve the expected clinical effect (McGrail et al., 2018; Jiang et al., 2021; McGrail, 2021). Thus, we are looking for a comprehensive prognostic index involving genetic mutations that can predict the efficacy of immunotherapy in r/m HNSCC.

In our study, we screened out three genes *PIK3CA*, *TP53*, and *ROS1*, and we constructed an ICI-related gene prognostic index TP-PR, together with TMB, for r/m HNSCC. The median OS of the high TP-PR group (TP-PR >0) was three times that of the low TP-PR group (TP-PR ≤0) (25 vs. 8 months). That means that patients with high TP-PR may benefit from ICI treatment than patients with low TP-PR. What is more, the high TP-PR group screened out more people who might benefit from immunotherapy than the TMB-H group. And TP-PR stratification had a higher AUC value than TMB stratification. Thus, we believe that TP-PR is a better predictor of the prognosis of r/m HNSCC immunotherapy than TMB alone. Our analysis is directly based on the cohort of r/m HNSCC patients who received immunotherapy. Unfortunately, we currently have no other cohort to verify this, which is our limitation.

Among the mutated genes contained in TP-PR, *PIK3CA* and *TP53* were common in patients with r/m HNSCC, while *ROS1* was relatively rare. But the mutation rate of *ROS1* in HNSCC in TCGA cohort is 5% (data not shown). Also, the *ROS1* mutation rate in r/m HNSCC in Morris and Allen’s cohorts is 5%. Thus, we believe *ROS1* is related to HNSCC and r/m HNSCC and might provide a direction for targeted drug selection in the future. Previously known HNSCC genes are *TP53*, *CDKN2A*, *PTEN*, *PIK3CA*, *HRAS*, *NOTCH1*, *IRF6*, *TP63*, etc. (Stransky et al., 2011). For r/m HNSCC, the known common mutated genes are *TP53*, *TERT*, *CDKN2A*, *PIK3CA*, *FAT1*, *NOTCH1*, etc. (Morris et al., 2017). Our study also showed that CDKN2A and NOTCH1 had high mutation rate in r/m HNSCC cohorts. However, CDKN2A or NOTCH1 mutation was not related to the prognosis of r/m HNSCC patients receiving immunotherapy. Thus, they were eliminated for further analysis.

Recent literature has shown that the combination of TMB and CNA or gene signature is more accurate than TMB alone in predicting ICI response. Patients with TMB-H and ADOBEC signature in breast cancer had sustained response to ICIs (Chumsri et al., 2020). Combining TMB and CNA can predict response and prognosis to immunotherapy in metastatic cancers or lung cancer (Liu et al., 2019; Xiang et al., 2020). In our study, *PIK3CA*, *TP53*, and *ROS1* were found to be associated with the OS in the ICI-treated cohort. The prognostic index that combined TMB with those gene mutations is supposed to better screen out groups who may benefit from immunotherapy.

Our study suggested that the differential gene expression in the high and low TP-PR groups mainly influenced BP related to development and metabolism, and metabolism-related signaling pathways. However, the pathway enrichment was analyzed in non-ICI cohorts, instead of ICI cohorts. Due to lack of relevant data, we did not have information on gene expression in the ICI cohorts and therefore could not obtain the results of pathway enrichment in the ICI cohorts. That is a limitation of our study. At present, there are only a few studies on this topic (Zhang et al., 2020b; Feng and Hess, 2021), and we hope that our study can act as a reference for follow-up research for identifying the underlying pathways and for seeking a possible link between enriched metabolic pathways and immunotherapy efficacy.

In fact, there may be some correlation between TMB and the expression of PD-L1, but this correlation is not present consistently (Luchini et al., 2019; Yarchoan et al., 2019). TMB is a more complex biomarker, which is related to tumor neoantigens and represents the whole immune microenvironment. Therefore, TMB has increasingly become a sensitive prognostic factor in immunotherapy. At present, TMB is mainly detected by second-generation sequencing (Klempner et al., 2020). Adjustments and improvements to standardize genetic testing offered by different manufacturer are needed, so that patients can get an accurate prediction of immunotherapy efficacy irrespective of the manufacturer.

Another limitation of our study is that we found no related data for human papillomavirus (HPV)-16 or other data for HPV status in the cohorts. But our findings are important for stratification of r/m HNSCC patients receiving immunotherapy. Future studies of HPV status and the efficacy of immunotherapy in r/m HNSCC patients will be interesting.

In conclusion, our study showed that TP-PR could act as a predictor of immunotherapy efficacy in r/m HNSCC, and patients with high TP-PR (TP-PR >0) had a better survival benefit than the patients with low TP-PR (TP-PR ≤0). We hope that our study will help in selecting the patient population for which immunotherapy is effective in order to better implement individualized treatment strategy with precision.

## Data Availability

The original contributions presented in the study are included in the article/Supplementary Material. Further inquiries can be directed to the corresponding author.
