# Supplementary figures and images for: Combination of Tumor Mutational Burden and Specific Gene Mutations Stratifies Outcome to Immunotherapy Across Recurrent and Metastatic Head and Neck Squamous Cell Carcinoma
Source: Front Genet. 2021 Nov 16;12:756506. doi: 10.3389/fgene.2021.756506 (PMC8637214; doi:10.3389/fgene.2021.756506)

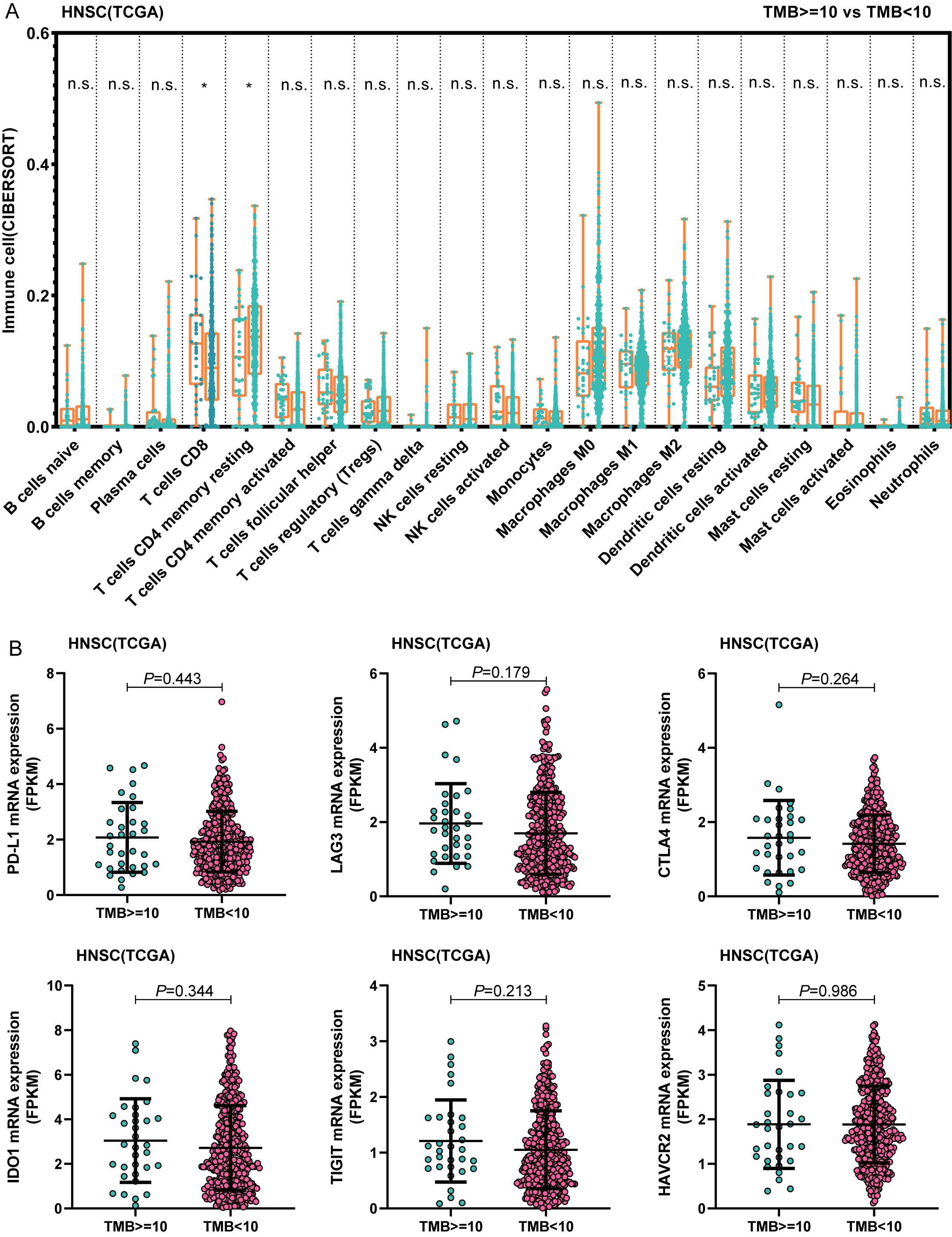

Supplement: Supplementary file 3 [file Image3.TIF]

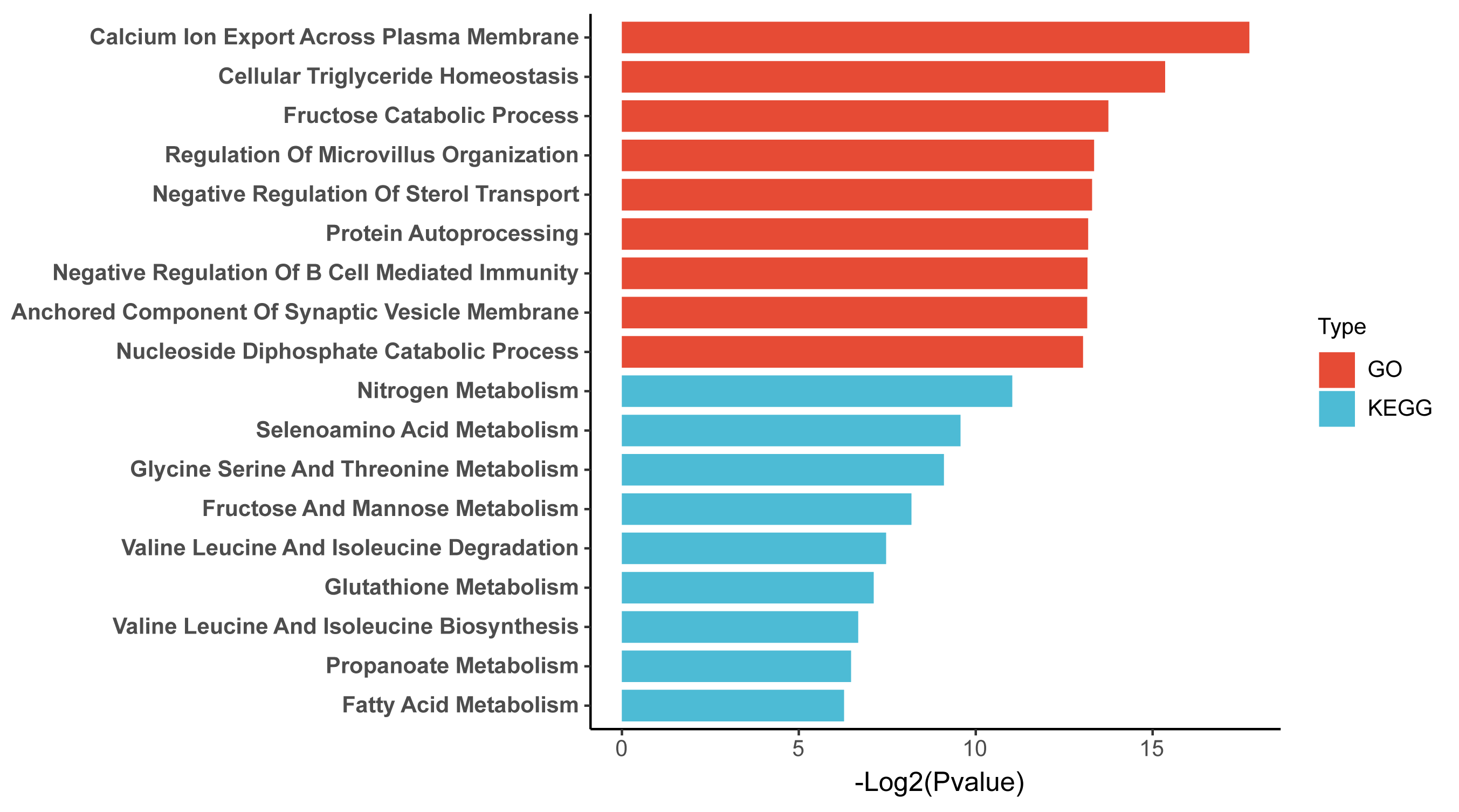

Supplement: Supplementary file 4 [file Image4.TIF]

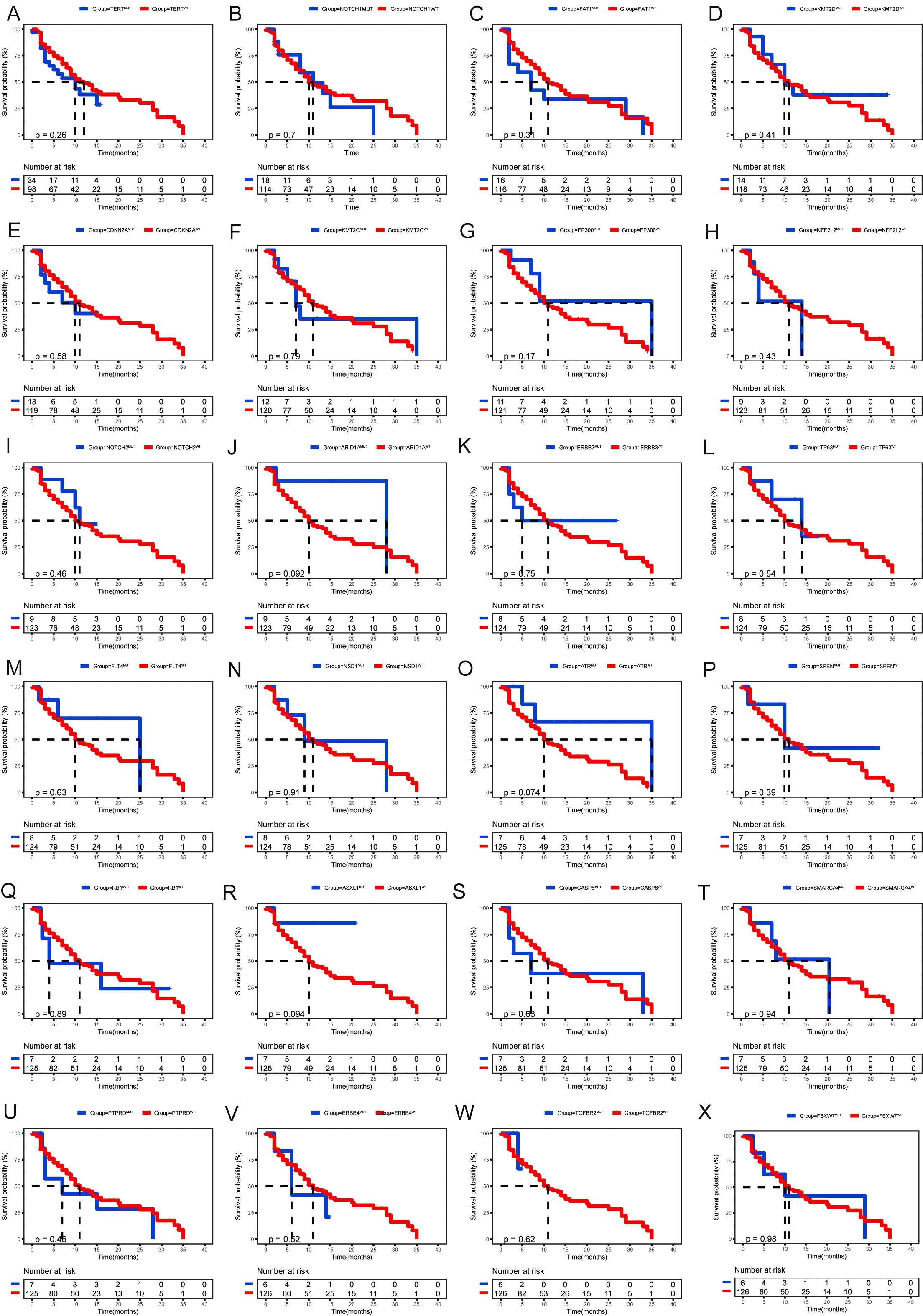

Supplement: Supplementary file 5 [file Image2.TIF]

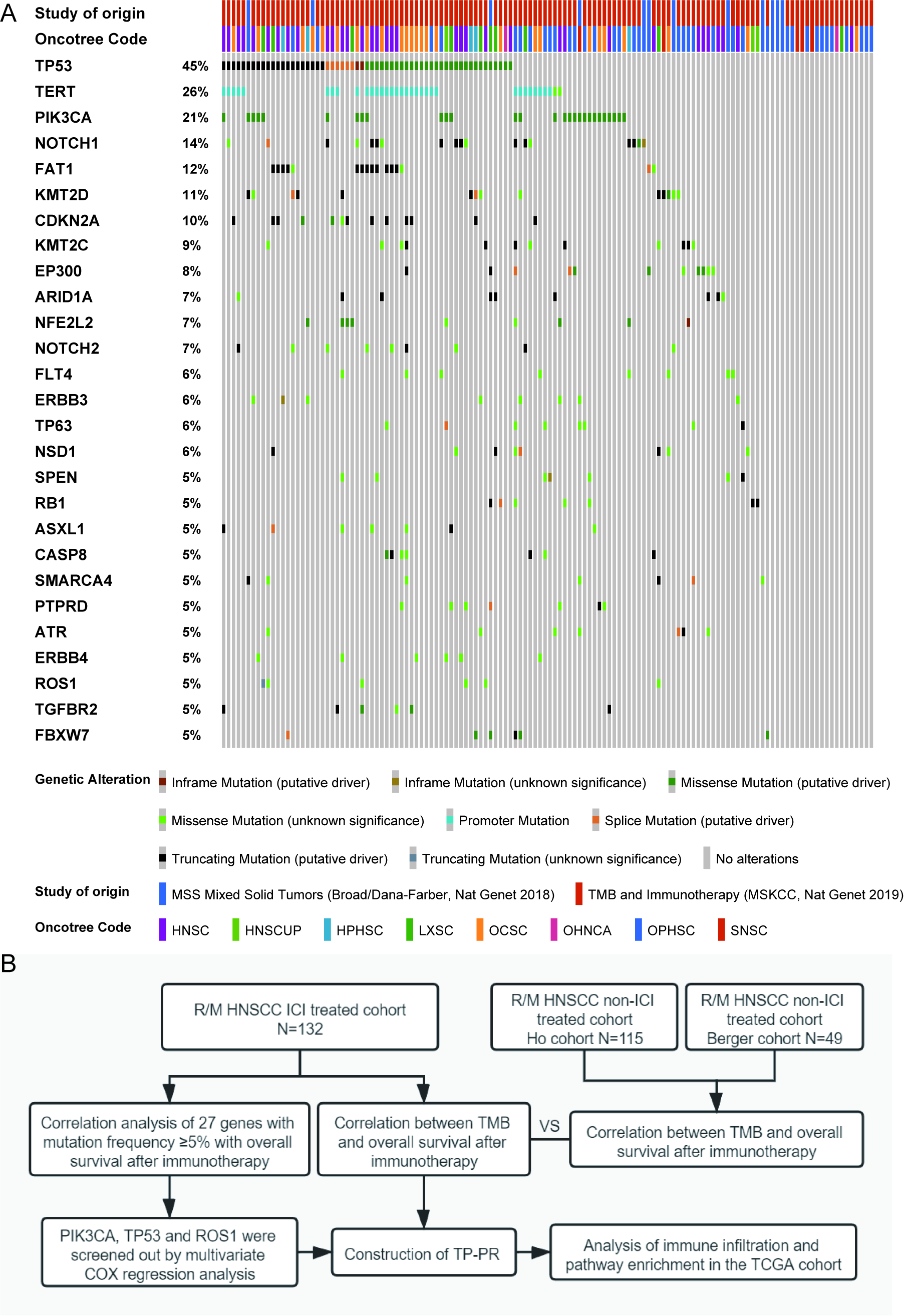

Supplement: Supplementary file 6 [file Image1.TIF]
